# Large-Fiber Neuropathy in Parkinson’s Disease: Clinical, Biological, and Electroneurographic Assessment of a Romanian Cohort

**DOI:** 10.3390/jcm8101533

**Published:** 2019-09-24

**Authors:** Oana Maria Vanta, Nicoleta Tohanean, Sebastian Pintea, Lacramioara Perju-Dumbrava

**Affiliations:** 1 Anatomy Department, University of Medicine and Pharmacy “Iuliu Hatieganu”, 400006 Cluj-Napoca, Romania; 2 Neurology Department, University of Medicine and Pharmacy “Iuliu Hatieganu”, 400012 Cluj-Napoca, Romania; nicoleta_alexa@yahoo.com (N.T.); lperjud@gmail.com (L.P.-D.); 3 Neurology I Department, Cluj-Napoca Emergency Clinical County Hospital, 400012 Cluj-Napoca, Romania; 4 Department of Psychology, Babes-Bolyai University, 40015 Cluj-Napoca, Romania; sebastianpintea@psychology.ro

**Keywords:** Parkinson’s disease, large-fiber neuropathy, L-Dopa, vitamin B12, folic acid

## Abstract

(1) Background: Increased attention has lately been given to polyneuropathy in Parkinson’s Disease (PD). Several papers postulated that large-fiber neuropathy (PNP) in PD is related to vitamin B12 deficiency and L-Dopa exposure. (2) Methods: Using a cross-sectional, observational study, we evaluated 73 PD patients without a previously known cause of PNP using clinical scores (UPDRS II and III and Toronto Clinical Scoring System), biological evaluation of vitamin B12 and folic acid, and nerve conduction studies to assess the prevalence and features of PNP. (3) Results: The prevalence of PNP was 49.3% in the study group. In the L-Dopa group, the frequency of PNP was 67.3% as compared to PNP in the non-L-Dopa group, where one subject had PNP (χ^2^ = 23.41, *p* < 0.01). PNP was predominantly sensory with mild to moderate axonal loss. Cyanocobalamin correlated with L-Dopa daily dose (*r* = −0.287, *p* < 0.05) and L-Dopa duration of administration (*r* = −0.316, *p* < 0.05). L-Dopa daily dose correlated with the amplitudes of sensory nerve action potentials of the superficial peroneal and radial nerves (*r* = −0.312, *p* < 0.05) (*r* = −0.336, *p* < 0.05), respectively. (4) Conclusions: PNP is more frequent in L-Dopa-treated patients than in L-Dopa-naïve patients. The results imply that longer exposure to high doses of L-Dopa may cause vitamin B12 and folate imbalance and PNP, secondarily.

## 1. Introduction

Parkinson’s disease (PD) is a neurodegenerative condition that affects the whole nervous system, but above all, the central nervous system [1,2]. Recent data suggest an impairment of the peripheral nervous system as well, which takes the form of large-fiber neuropathy (PNP) in L-Dopa-treated patients [3,4,5,6,7,8,9,10,11,12,13,14]. Hyperhomocysteinemia, low plasma levels of B12, B6, and folic acid, along with disease duration, age, and L-Dopa daily intake are considered to be the key factors in PD patients developing PNP [3,4,5,6,7,8,9,10,12,13,14,15,16]. Small-fiber neuropathy is considered to be an intrinsic feature of PD, mainly because of alpha-synuclein deposits at the level of sensory nerve fibers and modifications of intradermal nerve fiber density [17,18,19,20,21].

Several studies found that PNP prevalence was higher in PD than in age-matched controls, ranging from 15% to 73% in patients treated orally with L-Dopa [5,6,7,12,13,15,16], depending on the criteria used for the diagnosis of PNP. A recent review described an estimated prevalence of 30.2% after adjusting for case reports [22]. Data published on the prevalence of PNP in L-Dopa-naïve groups revealed a lower frequency of PNP when compared to L-Dopa-treated groups, ranging from 4.82% to 12.1% [7,15,23] and similar to the one in age-matched controls [9,12,15].

Contrary to these results, Shahrizaila et al. did not find any difference in PNP prevalence in L-Dopa versus L-Dopa-naïve patients (24% in the L-Dopa group versus 23% in the L-dopa-naïve group) [11].

Several papers described a positive relationship between higher L-Dopa daily intake and the presence of PNP [7,9,10,12,13,14,15,24]. Age was found to be a risk factor for PNP in PD [12,13]. Ceravolo et al. found that the risk for PNP increases by 7% for each year of age and demonstrated a threefold increase in risk for subjects with more than 3 years of exposure to L-Dopa as compared to no or short exposure [15]. The duration of PD was also associated with the presence of PNP [13,14,15].

PNP in PD seems to be caused by underlying metabolization pathways of L-Dopa [6,25,26]. The conversion of L-Dopa to dopamine leads to homocysteine (Hyc) formation. The remethylation of homocysteine requires either vitamin B12 or, by using another pathway, methylenetetrahydrofolate and vitamin B6 [27]. Consequently, chronic L-Dopa intake determines elevated Hyc levels, vitamin B12, B6, and folate deficiency [25].

Evaluation of Hyc, methylmalonic acid, and vitamin B12 showed that PD patients with PNP have significantly higher levels of Hyc [3,6,7,12,13,14,24,28], methylmalonic acid [8,12,14] and borderline or lower vitamin B12 values [6,10,13,14,15] than patients without PNP.

Hyperhomocysteinemia is known to appear in PD patients undergoing L-Dopa therapy [29,30], and several reports found that the association of Cathecol-O-Methyltransferase inhibitors (COMT-I) can decrease plasmatic Hyc [31,32,33]. Hence, the potential beneficial effect of associating COMT-I to L-Dopa to prevent PNP has been demonstrated previously [34].

In 2004, Muller et al. described a relation between high Hyc plasma levels and axonal degeneration of the sural nerve in L-Dopa-treated patients [35]. In a series of cases with PNP in PD, sural nerve biopsies revealed primary axonal degeneration with secondary demyelination [36].

In orally treated PD patients, the electroneurographic assessment revealed a chronic distal, symmetrical, axonal predominant sensory type of polyneuropathy [13,22,37].

A series of case reports were published, describing severe acute or subacute axonal forms of PNP with or without demyelinating features, in PD patients undergoing L-Dopa/Carbidopa intestinal gel infusion (LCIG) with vitamin B12, B6 deficiencies or elevated Hyc [27,38,39,40,41,42]. A recent review on this topic depicted an estimated PNP prevalence of 42.1% in LCIG groups, after excluding case reports and case series, considering that the majority of cases were sensory axonal—90.8% and 35.7% of published cases had an acute or subacute onset [22]. Cross-sectional studies described a higher prevalence of PNP in LCIG-treated patients, when compared to the oral L-Dopa group [5,6,7,28], and found correlations to lower pyridoxine plasmatic values [6], higher Hyc plasmatic values [6,7,28], and L-Dopa daily dose [5,7].

Electroneurographic examinations detected a more severe axonal loss [6] and a higher number of neurographically impaired nerves [5] in subjects undergoing LCIG than in orally treated ones.

Several authors suggested that not only high doses and more prolonged exposure to L-Dopa in LCIG groups may determine PNP, but also the form of administration. The direct administration of the gel in the jejunum may alter the intestinal homeostasis and absorption of vitamins and other nutrients [5,25,27,43,44].

More importantly, a recent study suggested—after adjusting for age, disease duration, and L-Dopa daily dose—that PNP is associated with worse axial motor impairment, nonmotor, and autonomic features, and implied that PNP might represent a marker of a severe PD phenotype [24].

In light of this data, the present report aims to establish the frequency, clinical, and electroneurographic characteristics of PD patients with large-fiber neuropathy from a center in Romania.

## 2. Materials and Method

In this cross-sectional, observational study, we included patients that suffer from PD according to the UK Parkinson’s Disease Society Brain Bank [45] and newly reviewed criteria by the Movement Disorders Society [46] with and without L-dopa treatment. The subjects were admitted to the Neurology I Department of the County Emergency Clinical Hospital in Cluj-Napoca, a general neurology department, from January 2017 to June 2019.

Patients with previously known hereditary, metabolic (including diabetes mellitus), toxic, inflammatory, or autoimmune causes for PNP were excluded. In addition, patients with entrapment neuropathies, moderate and severe radiculopathies, plexopathies, or having undergone spinal surgery or gastric resection were not included. Patients known to have Biermer anaemia undergoing treatment or those over the age of 80 were also excluded.

### 2.1. Clinical Features

All patients underwent a complete neurological examination using The Unified Parkinson’s Disease Rating Scale (UPDRS ) part II and III for evaluation of motor symptoms and the Toronto Clinical Scoring System (TCSS) [47] for the clinical assessment of peripheral nerve involvement. TCSS is a validated tool for the clinical diagnosis of polyneuropathy in diabetes mellitus, with a cut-off value above 6 points. The TCSS also asseses the severity of polyneuropathy and has been used in other similar studies [5,7,12,13,28].

The progression of PD was evaluated using the Hoehn and Yahr scale. Patients’ charts were reviewed for PD using the following criteria: disease duration, L-Dopa daily dose (LDD) over the last three months, and duration of administration of L-Dopa (LDDA). Other dopaminergic drugs were not considered and the L-Dopa equivalent dose was not calculated.

### 2.2. Biological Features

Prior to enrollment in the study, all potential subjects were screened for possible causes of polyneuropathy—a jeun glycemia, red and white blood cells, renal function, liver enzymes, electrolytes, protein electrophoresis, and thyroid function. For all the enrolled subjects, seric folic acid and cyanocobalamin levels were dosed, and a jeun seric glucose was repeated. Tests for homocysteine, vitamin B6, and methylmalonic acid were not available in our laboratory and, consequently, were not conducted. Subjects having two repeated fasting seric glucose levels above 125 mg/dL were not included. The hospital laboratory worked-up blood samples on a UniCel DxI 600 analyzer (Beckman Coulter Inc., Brea, California, CA, USA). The process is automatic chemiluminescence. Reference values, according to the laboratory, were 180–914 pg/mL for vitamin B12 and 5.9–23.2 ng/mL for folic acid, respectively.

### 2.3. Nerve Conduction Studies

Based on the fact that large-fiber polyneuropathy in PD is considered to be distal and symmetrical, predominantly axonal, and sensory–motor, affecting primarily, the sensory fibers [37] resembling the polyneuropathy in diabetes mellitus, all patients underwent electroneurographic assessment (ENoG) on a Keypoint Medtronic machine using surface electrodes. All patients had unilateral assessment of the sural, superficial peroneal, tibial, and common peroneal nerves. The following parameters were obtained for each sensory nerve: amplitude of sensory action potential (aSNAP) and sensory nerve conduction velocity (sNCV) of the sural and superficial peroneal nerve. For motor nerves, tibial, and common peroneal nerves, the distal motor latency (DL), the amplitude of compound muscle action potential (aCMAP), and the motor nerve velocity (mNCV) were obtained. The sural and tibial nerves were examined bilaterally to assess the symmetry of the parameters. For most of the subjects, the sensory response and sensory nerve velocity of the radial nerve and motor and sensory response of the median nerve (unilaterally) were also evaluated to assess the extent of a possible axonal loss, if impaired values were found in the lower limbs. The examination was made in optimal environmental conditions—a warm room with a skin temperature over 33 °C. All obtained values were adjusted for height and weight. All sensory nerve evaluations were carried out in antidromic conditions, with the stimulation point being situated at a distance of 12 to 14 cm proximal to the recording site, as stated in the handbook [48], and the average aSNAP and sNCV was recorded and taken into account.

Axonal loss is considered to be present when the aSNAP for sensory fibers or aCMAP for motor nerves is reduced, but with normal velocities (above 70% of the lower normal limit (LNL)) and mild or no prolongation of motor or sensory distal latency (less than 125% of the upper normal limit (UPL)) [48]. Demyelinating features were considered if reduced velocities or prolonged distal latencies were observed.

**Figure 1:**
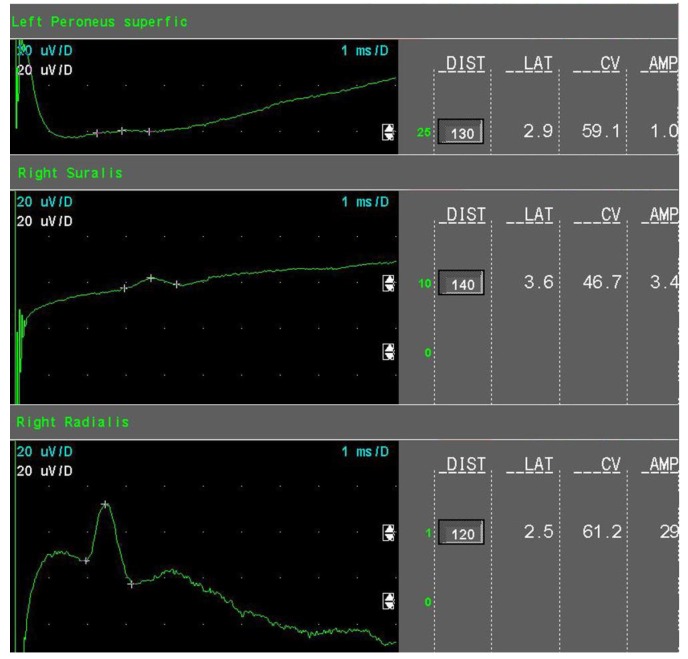
Sensory nerves conduction studies in mild sensory axonal loss (example).

**Figure 2:**
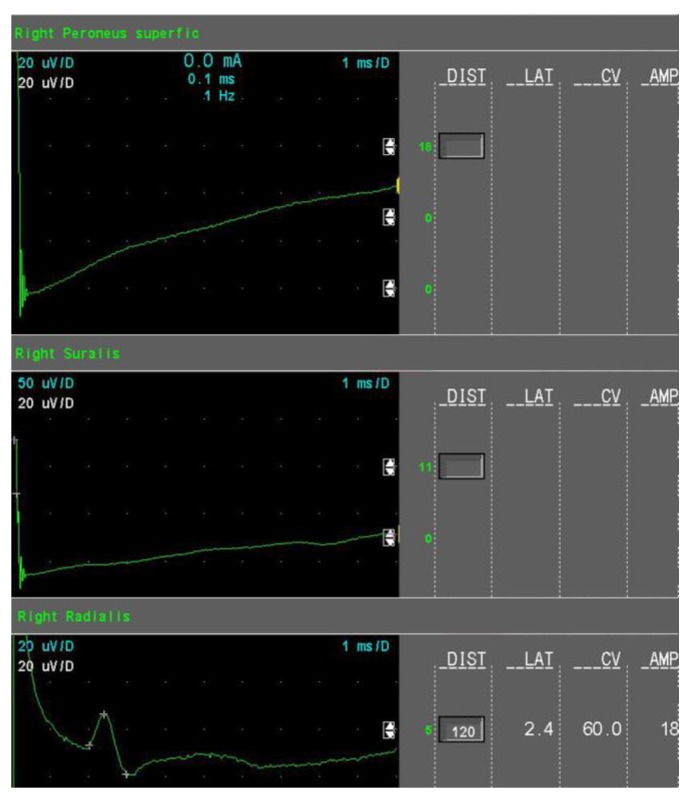
Sensory nerves conduction studies in moderate sensory axonal loss (example).

**Figure 3:**
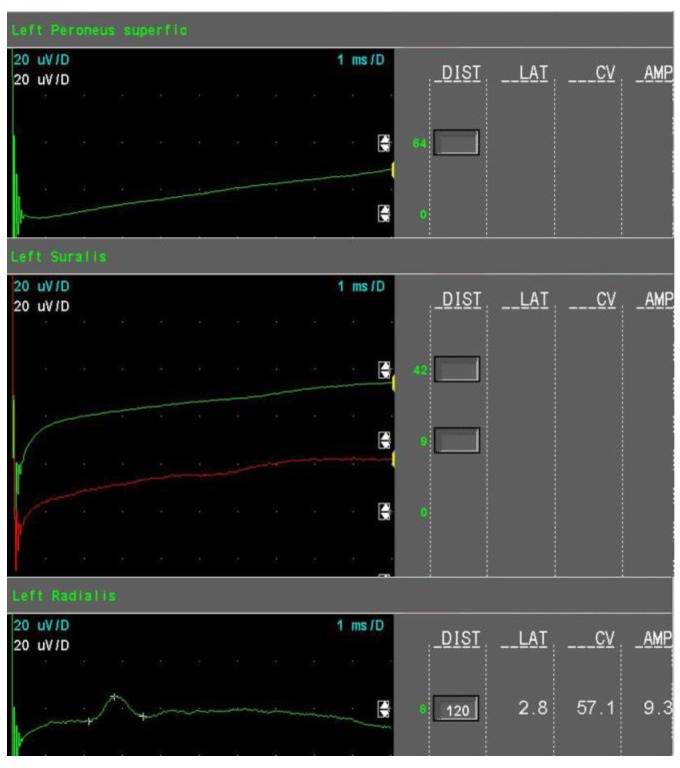
Sensory nerve conduction studies in severe sensory axonal loss (example).

The amount of axonal loss was quantified as follows:

- Mild sensory axonal loss: a reduction of the SNAP amplitude of more than 15% but no more than 50% of LNL for the sural and superficial peroneal nerves. LNL values for the sural and superficial peroneal nerves were considered to be 6 µV and 20 µV for the radial nerve, respectively (Figure 1).
- Moderate sensory axonal loss: a reduction of the SNAP amplitude of more than 50% of LNL for the sural and superficial peroneal nerves and a decrease of the SNAP amplitude of more than 15% but no more than 50% of LNL for the radial nerve (Figure 2).
- Severe sensory axonal loss: a reduction of the SNAP amplitude of more than 50% of LNL for sural, superficial peroneal nerve, and radial nerves (Figure 3).
- Motor axonal loss: a reduction of the CMAP amplitude of more than 25% of LNL for the tibial nerve (LNL = 4 mV) or/and the peroneal nerve (LNL = 2 mV), or/and the median nerve (LNL = 4 mV).

One impaired value of the SNAP or CMAP amplitude or one unexcitable sensory nerve were not criteria for axonal loss.

According to these ENoG changes, the following subtypes of neuropathy that can be found in the group, from an electrodiagnostic point of view, were established:

1. Mild axonal sensory polyneuropathy—the presence of mild sensory axonal loss, absence of motor axonal loss, and demyelinating features;
2. Moderate axonal sensory PNP—the presence of moderate sensory axonal loss, absence of motor axonal loss, and demyelinating features;
3. Severe axonal sensory with motor features—the presence of severe sensory axonal loss and motor axonal loss with or without demyelinating features.

A PD patient was considered to have PNP if they met the criteria of likelihood, as stated by J.D England et al. in the special article for AAN on “Distal symmetric polyneuropathy: a definition for clinical research” [49].

### 2.4. Statistical Analyses

Descriptive statistics for normally distributed continuous variables (e.g., age) were presented as a mean ± standard deviation. The statistical significance of differences between two independent groups was tested using the independent samples *t*-test, while comparisons between three groups were performed using the Analysis of Variances (ANOVA) followed by Scheffe post-hoc tests to identify the pairs of groups with significant differences. Correlations between variables were assessed using the Pearson correlation coefficient.

Descriptive statistics for categorical variables (e.g., gender) were presented as counts and proportions, and for statistical comparisons between groups, the Chi-square test was used. For all inferential analysis, a two-sided *p*-value <0.05 was considered statistically significant.

Statistical analysis was performed using the SPSS 20.

Signed informed consent was obtained for each patient. This research was carried out under the Helsinki Declaration.

## 3. Results

Seventy-three subjects (50.7% males, mean age 65.44 ± 9.87) were enrolled according to the inclusion and exclusion criteria stated above. The prevalence of PNP in the entire group was 49.3%. There was an association between B12 plasma deficit (less than 180 pg/mL) and PNP (χ^2^ (1, 73) = 15.62, *p* < 0.001), and folic acid plasma deficit (less than 5.9 ng/mL) and PNP (χ^2^ (1, 73) = 10.83, *p* < 0.001).

The aSNAP of the sural nerve correlated with the vitamin B12 seric value (*r* = 0.444, *p* < 0.01) and folic acid (*r* = 0.314, *p* < 0.01) and the aSNAP of the superficial peroneal nerve correlated with the B12 plasmatic value (*r* = 0.349, *p* < 0.01) and folic acid (*r* = 0.347, *p* < 0.01), while the aSNAP of the radial nerve correlated only with cyanocobalamin (*r* = 0.375, *p* < 0.01). The TCSS correlated positively with UPDRS part II (*r* = 0.314, *p* < 0.01), UPDRS part III *(r* = 0.307, *p* < 0.01), and age (*r* = 0.429, *p* < 0.01).

### 3.1. L-Dopa Group Versus Non-L-Dopa Group

In an attempt to analyze the differences between L-Dopa-treated patients and those without L-Dopa, the group was separated into two subgroups—the L-Dopa group, which consisted of 52 subjects receiving L-Dopa on a daily basis, and the 21 L-Dopa-naïve subjects (the non-L-Dopa group) on other dopaminergic therapies. Demographic, clinical, biological features and differences between the L-Dopa group and the Non-L-Dopa group are summarized in Table 1. Relevant nerve conduction studies results are represented in Table 2.

The frequency of PNP in the L-Dopa group was 67.3% (35 subjects) as compared to PNP in the non-L-Dopa group, where one subject fulfilled the criteria for PNP (χ^2^ = 23.41, *p* < 0.01). Nerve conduction studies revealed a predominant sensory axonal loss.

In the L-dopa group, the aSNAP of the sural nerve correlated with the vitamin B12 seric value (*r* = 0.401, *p* < 0.01) and folic acid (*r* = 0.329, *p* < 0.05) and the aSNAP of the superficial peroneal nerve correlated with the B12 plasmatic value (*r* = 0.405, *p* < 0.01) and folic acid (*r* = 0.430, *p* < 0.01), while the aSNAP of the radial nerve correlated only with cyanocobalamin (*r* = 0.327, *p* < 0.05). Cyanocobalamin inversely correlated with the LDD (*r* = −0.287, *p* < 0.05) and the LDDA (*r* = −0.316, *p* < 0.05). The LDD negatively correlated with the aSNAP of the superficial peroneal nerve and the aSNAP of the radial nerve (*r* = −0.312, *p* < 0.05) (*r* = −0.336, *p* < 0.05), respectively. The LDDA correlated with the aSNAP of the superficial peroneal nerve (*r* = −0.305, *p* < 0.05) and of the radial nerve (*r* = −0.357, *p* < 0.05) and the sensory nerve conduction velocity (NCV) of the superficial peroneal nerve (*r* = −0.476, *p* < 0.05) and of the radial nerve (*r* = −0.315, *p* < 0.05).

### 3.2. Differences Between the L-Dopa-PNP Group, the L-Dopa-Non-PNP Group Versus the Non-L-Dopa-Non-PNP Group

Further, due to the heterogeneity of the two groups regarding age, PD duration, motor impairment, and ENoG parameters, the following three subgroups were formed:

1. Subgroup 1—L-Dopa-PNP—included 35 patients undergoing L-dopa treatment with polyneuropathy;
2. Subgroup 2—L-Dopa-non-PNP—included 17 patients undergoing L-dopa treatment without polyneuropathy;
3. Subgroup 3—non-L-Dopa-non-PNP—included 20 patients without L-dopa treatment and polyneuropathy.

Subgroup 1 did not differ significantly from subgroup 2 concerning age, motor impairment, and activities of daily living. The LDD and LDDA were considerably higher in subgroup 1 than in subgroup 2 (984.23 ± 441.94 vs. 702.06 ± 317.15, *p* < 0.05), (80.57 ± 54.22 vs. 55.41 ± 34.17, *p* = 0.08), respectively. Cyanocobalmin and folate seric values were significantly lower in subgroup 1 than subgroup 2 (198.37 ± 100.24 vs. 291.47 ± 103.63, *p* = 0.003), (6.81 ± 3.91 vs. 10.77 ± 5.36, *p* = 0.007). More characteristics of the subgroups and differences are reported in Table 3.

### 3.3. The Severity of Axonal Loss in the L-Dopa-PNP Group

Based on the ENoG and the criteria stated above for axonal loss, from the 35 subjects included in the L-Dopa-PNP group, 10 (28.57%) showed modifications compatible with mild sensory axonal neuropathy, 10 (28.57%) had moderate sensory axonal PNP, and 15 showed severe sensory axonal loss and axonal motor loss (*p* = 0.615). Details on the ENoG parameters for every subtype of PNP are summarized in Table 4.

ANOVA analysis in the three severity PNP subgroups revealed differences regarding PD in terms of the duration (6.8 ± 4.1 vs. 8.85 ± 5.00 vs. 11.2 ± 5.25 years, *p* = 0.099), LDD (778.75 ± 167.5 vs. 1045.00 ± 469.75 vs. 1080.70 ± 521.22 mg/day, *p* = 0.221), and LDDA (85.20 ± 41.67 vs. 67.80 ± 36.01 vs. 104.00 ± 64.25 months, *p* = 0.076), but not age (67.00 ± 8.89 vs. 68.80 ± 7.64 vs. 69.00 ± 8.19, *p* = 0.823). However, none of these differences met the criteria for statistical significance.

## 4. Discussion

The prevalence of PNP in the studied PD group was 49.3%, and it resembled that described in some recently published studies [3,4,13,14] but was higher than in others [9,10]. The prevalence of PNP in the L-Dopa group was 67.3%, similar to two other studies that compared neuropathy in PD patients undergoing LCIG and oral administration [5,6].

The mean age of patients with associated PNP was 68 years old, similar to the age of the subjects with PNP in research conducted by Jugel et al. [5]. However, the subjects featured in the present work were younger than those included in other studies [8,12,13,14,15]. Age was found to correlate to certain sensory ENoG parameters, as expected because the amplitude of sensory nerve action potentials decreases with age [48].

Limits of this study include the small sample size, lack of a control group, and the single-center basis of the study. Other restrictions are the lack of fasting homocysteine, methylmalonic acid, and pyridoxine that other studies evaluated and determined to be a positive correlation between hyperhomocysteinemia and PNP [3,5,6,7,12,13,14,24,43]. Also, the occurrence of small-fiber neuropathy was not assessed as this was not our primary objective.

One recent study found that low B6 plasma values correlated to PNP and L-Dopa doses [6]. Recently, Cossu et Melis postulated that prolonged L-Dopa administration, due to its metabolic conversion to dopamine, modifies the peripheral nerve homeostasis by determining homocysteine accumulation and depletion of pyridoxine, folate, and vitamin B12, secondarily [25].

A significant association between cobalamin and folate deficiency with PNP was determined in this study (χ^2^ = 15.62, *p* < 0.001 and χ^2^ = 10.83, *p* = 0.001, respectively). Several studies also determined a positive relationship between lower plasma levels of cyanocobalamin and PNP in PD [10,14,15,36,50]. Thus far, only a single published study has demonstrated that lower plasma levels of folic acid, less than 10 µg/L, are linked to PNP in PD [9], while other studies failed [3,7,12,13,14].

To our knowledge, this is the first study to assess axonal sensory loss at the level of the radial nerve in PD patients, also correlating the ENoG parameters to clinical and biological features. From what we know, this is the first study to assess neuropathy in Romanian PD patients.

Muller et al., in 2004, found a significant correlation between the SNAP amplitude of the sural nerve and increased levels of homocysteine in PD patients undergoing long-term L-Dopa therapy [35]. In the present study, significant correlations between the aSNAP of the sural nerve and the superficial peroneal nerve to vitamin B12 and folate were found. Furthermore, the aSNAP of the radial nerve described the same trend of association with cobalamin, but B12 and folic acid deficits are a known treatable cause of neuropathy [9]. This positive correlation suggests that higher plasma values of vitamin B12 and folic acid may even have a protective role.

The aSNAP of the superficial peroneal and radial nerves negatively correlated to daily intake and exposure to L-Dopa, suggesting that L-Dopa may affect axons, hence the axonal sensory loss. This possible relation was undermined by the correlation of vitamin B12 to the LDD and LDDA. These results imply that prolonged exposure to high doses of L-Dopa can be associated with vitamin B12 and folate imbalance and, secondarily, with PNP.

The severity of axonal sensory and motor polyneuropathy is usually assessed by ENoG at the level of lower limbs, but the pattern of this neuropathy is “length-dependent” [37]. Impaired values are found initially at the level of nerves in the lower limbs and, as axonal loss progresses, nerves in the arms are affected [48,49]. The Sural–Radial SNAP amplitude ratio can be used to evaluate mild sensory axonal neuropathy in PD [37,51]. Although the radial nerve in almost all subjects was assessed, this ratio was not calculated. However, correlations that imply that the severity of PNP in PD is related to L-Dopa daily intake and prolonged exposure were found. This implication was also found by Toth et al. in 2008 and 2010 [12,14] when they revealed a higher L-Dopa equivalent daily dose in the PNP group compared to the non-PNP group, that positively correlated to PNP severity, as assessed by the TCSS [12]. Age and PD duration contribute indirectly to the severity of PNP in PD. Nonetheless, a clear link between PD duration, age, and PNP in PD, relations that were albeit revealed in other published studies [6,7,8,9,15,52], could not be established in the present work. On the other hand, the risk for PNP was not stratified, in contrast to the study of Ceravolo et al. [15].

In a recently published trial, the authors did not report any cases in which PNP developed as an adverse effect of L-Dopa therapy, when the investigators followed up patients with early PD who took under 300/75 mg L-Dopa/Carbidopa a day for 80 weeks [53].

Bearing in mind that PNP in PD can affect motor functions and that L-Dopa therapy remains the standard therapy in PD, improving motor functions and quality of life, screening for vitamin B12 and folic acid deficiencies should be taken into consideration by any clinician when treating a PD patient. Furthermore, correcting those deficiencies is mandatory, and prevention may be taken into account even if it is still debatable, at least regarding vitamin B6 due to its interactions with decarboxylase inhibitors [25].

## 5. Conclusions

The reported results strongly suggest that large-fiber neuropathy in PD is common in L-Dopa-treated patients. The axonal injury is predominantly sensory and mainly mild to moderate. PNP in PD seems to be primarily related to L-Dopa treatment, correlating to the L-Dopa daily dose and longer exposure, and secondarily to cobalamin and folate deficiency that derives from L-Dopa metabolism.

Further long-term prospective studies on larger cohorts are needed for a possible task force to be able to recommend evidence-based strategies for the prevention or appropriate treatment of large-fiber neuropathy in PD.

## Figures and Tables

**Table 1:** Demographic, clinical, and biological features of the groups (independent samples *t*-test/Chi-square test). LDD, L-Dopa Daily Dose; LDDA, L-Dopa Duration of administration. Results are represented as mean (SD). F, Female; M, Male.

	L-Dopa Group (*n* = 52)	Non-LDopa Group (*n* = 21)	*p*
Age (years)	67.60 (8.58)	60.10 (11.02)	0.001
Sex (F:M)	26:26	10:11	0.853
Hoehn and Yahr stage	3.19 (0.72)	2.05 (0.59)	0.001
PD Disease Duration(years)	8.65 (5.17)	2.31 (1.52)	0.001
UPDRS II	15.38 (9.15)	6.05 (3.46)	0.001
UPDRS III	21.62 (10.06)	10.62 (4.67)	0.001
TCSS	8.46 (5.3)	3.43 (3.28)	0.001
Cyanocobalmin (pg/mL)	228.81 (109.60)	306.38 (160.47)	0.020
Folic acid (ng/mL)	8.1 (4.77)	9.05 (3.46)	0.413
LDD (mg/day)	891.98 (423.827)	-	-
LDDA (months)	72.34 (49.68)	-	-

**Table 2:** Electrophysiological parameters in the two groups (independent samples *t*-test). Results are represented as mean (SD). ENoG, electroneurographic assessment; NCV, nerve conduction velocity.

ENoG Parameters	L-Dopa Group (*n* = 52)	Non-L-Dopa Group (*n* = 21)	*p*
aSNAP Sural nerve (µV)	5.32 (4.64)	9.99 (3.91)	0.001
Sensory NCV Sural nerve (m/s)	50.83 (9.64)	52.75 (7.52)	0.424
aSNAP Superficial peroneal nerve (µV)	2.88 (3.44)	8.00 (3.69)	0.001
Sensory NCV Superficial peroneal nerve (m/s)	47.58 (8.48)	48.64 (7.84)	0.656
aSNAP Radial nerve (µV)	16.72 (8.42)	21.94 (4.99)	0.004
Sensory NCV Radial nerve (m/s)	59.2 (10.74)	66.47 (6.99)	0.011
aSNAP Median nerve (µV)	13.08 (6.53)	36.33 (13.2)	0.001
Sensory NCV Median nerve (m/s)	55.21 (12.56)	63.03 (10.09)	0.324
aCMAP Tibial nerve (mV)	7.69 (3.44)	8.89 (2.95)	0.165
aCMAP Median nerve (mV)	6.04 (1.84)	7.87 (1.82)	0.031
aCMAP Common peroneal nerve (mV)	4.75 (2.79)	6.66 (2.48)	0.008
Motor NCV Common peroneal nerve (m/s)	49.29 (7.24)	50.14 (4.23)	0.541

**Table 3:** Results for the Analysis of Variances (ANOVA) and Scheffe post-hoc analyses (pairs of groups with significant differences). Results are represented as mean (SD). PD, Parkinson’s disease; LDD, L-Dopa daily dose; LDDA, L-Dopa duration of administration; TCSS, Toronto Clinical Scoring System; PNP, Large-fiber neuropathy.

	L-Dopa-PNP *n* = 35	L-Dopa-non-PNP *n* = 17	non-L-Dopa-non-PNP *n* = 20	*F*	*p*	*Post-hoc Scheffe*
Age (years)	68.37 (8.05)	66.00 (9.64)	60.85 (10.73)	4.23	0.018	1–3
Hoehn and Yahr stage	3.31 (0.64)	2.94 (0.83)	2.05 (0.60)	22.08	0.001	1–3, 2–3
Cyanocobalmin (pg/mL)	198.37 (100.24)	291.47 (103.63)	307.45 (164.56)	6.32	0.003	1–2, 1–3
Folic acid (ng/mL)	6.81 (3.91)	10.77 (5.36)	8.85 (3.42)	5.39	0.007	1–2
PD Disease Duration (years)	9.27 (5.09)	7.38 (5.24)	2.13 (1.3)	16.74	0.001	1–3, 2–3
LDD (mg/day)	984.23 (441.94)	702.06 (317.15)	-	5.52	0.023	1–2
LDDA (months)	80.57 (54.22)	55.41 (34.17)	-	3.05	0.087	1–2
UPDRS II	16.2 (8.53)	13.17 (10.4)	6.25 (3.42)	9.95	0.001	1–3, 2–3
UPDRS III	22.2 (9.24)	20.41 (11.77)	11.05 (4.35)	10.37	0.001	1–3, 2–3
TCSS	10.31 (4.93)	4.65 (3.87)	3.1 (2.99)	21.82	0.001	1–2, 1–3

**Table 4:** Descriptive statistics of electroneurographic assessment (ENoG) parameters in the severity subgroups. N.V., normal values according to the laboratory; aSNAP, amplitude of sensory action potential; aCMAP, amplitude of compound muscle action potential. Results are represented as mean (SD).

ENoG Parameters	Mild Axonal Sensory PNP *n* = 10	Moderate Sensory Axonal PNP *n* = 10	Severe Sensory Axonal PNP with Axonal Motor Loss *n* = 15
aSNAP Sural nerve (µV) (N.V.^1^ > 6) (unobtained in 8 subjects)	4.43 (0.73)	2.14 (1.805)	1.66 (1.731)
Sensory NCV Sural nerve (m/s) (N.V. > 40)	54.64 (9.691)	51.21 (14.449)	46.23 (9.884)
aSNAP Superficial peroneal nerve (µV) (N.V. > 6) (unobtained in 24 subjects)	1.76 (1.985)	0.28 (0.533)	0.29 (0.605)
Sensory NCV Superficial peroneal nerve (m/s) (N.V. > 40)	42.38 (3.757)	51.1 (7.882)	43.1 (21.140)
aSNAP Radial nerve (µV) (N.V. > 20)	21.53 (7.627)	18.27 (3.959)	8.82(5.944)
Sensory NCV Radial nerve (m/s) (N.V. > 50)	60.54 (5.135)	62.19 (10.385)	55.49 (13.689)
aSNAP Median nerve (µV) (N.V. > 10)	18.15 (5.502)	20.5 (0.707)	7.21(3.061)
Sensory NCV Median nerve (m/s) (N.V. > 50)	55.05 (8.878)	69.00 (18.385)	49.89 (14.189)
aCMAP Tibial nerve (mV) (N.V. > 4)	9.36 (3.162)	8.78 (3.063)	5.01 (3.574)
Motor NCV Tibial nerve (m/s) (N.V. > 40)	46.74 (4.599)	51.43 (7.501)	49.71 (9.193)
aCMAP Median nerve (mV) (N.V. > 4)	6.52 (1.825)	4.30 (0.00)	5.54 (2.148)
Motor NCV Median (m/s) (N.V. > 50)	53.87 (3.629)	65.60 (0.00)	53.93 (6.823)
aCMAP Common peroneal nerve (mV) (N.V. > 2)	4.69 (1.92)	6.14 (2.348)	2.67 (2.603)
Motor NCV Common peroneal nerve (m/s) (N.V. > 40)	50.07 (8.94)	46.41 (4.309)	49.11 (6.562)

^1^ N.V.—normal values according to the laboratory.
